# Effect of local aromatase inhibition in endometriosis using a new chick embryo chorioallantoic membrane model

**DOI:** 10.1111/jcmm.14372

**Published:** 2019-06-14

**Authors:** Nicola Pluchino, Giorgia Poppi, Lucile Yart, Roberto Marci, Jean‐Marie Wenger, Jean‐Christophe Tille, Marie Cohen

**Affiliations:** ^1^ Division of Obstetrics and Gynecology University Hospital of Geneva Geneva Switzerland; ^2^ Department of Morphology, Surgery and Experimental Medicine University of Ferrara Ferrara Italy; ^3^ Division of Pathology University Hospital of Geneva Geneva Switzerland

**Keywords:** anastrozole, chick embryo chorioallantoic membrane, endometriosis

## Abstract

Endometriosis is an oestrogen‐dependent, inflammation‐driven gynaecologic disorder causing severe disability. Endometriosis implants are characterized by unbalanced local oestrogen metabolism leading to hyperoestrogenism and aromatase up‐regulation is one of main mechanism involved. Aromatase inhibitors such as letrozole or anastrozole use in young women are associated with severely side effects limiting their long‐term clinical use. An endometriosis‐targeted inhibition of local aromatase could be a viable alternative, although the role of the local inhibition of this enzyme is still unclear. Using a new chick embryo allantoic membrane (CAM) model incorporating xenografted human endometriosis cyst, we showed that topical treatment with anastrozole reduced lesion size, although oestrogens produced by CAM female embryo blunted this effect. Xenografted human endometriosis CAM is a new efficient model for the screening of new drugs targeting endometriosis tissue.

## 1 INTRODUCTION

Endometriosis is a condition that has long plagued women and baffles the medical world on its development and causative factors.^1^ The disease is exemplified in the development of the endometrial glands and stroma outside the uterine cavity Endometriosis usually presents non‐malignant, however, ectopic endometrial tissue and resultant inflammation can be severely debilitating.^2^

Endometriosis implants are characterized by unbalanced local oestrogen metabolism leading to hyperoestrogenism.^3^, ^4^ Aromatase is one key enzyme in the biosynthesis of oestrogens. It is mainly found in the ovarian granulosa cells, and in a lesser extend in the adipose tissue, brain, bone and placenta. High aromatase expression has been also observed in the eutopic endometrium as well as in endometriosis implants.^5^ This enzymatic imbalance is thought to raise oestrogen activity into the endometriotic lesion, maintaining the loop between local hyperoestrogenic state, inflammation and proliferation and survival of endometriotic implants.^6^ Endometriosis current treatment is thus mainly based on suppression of oestrogen production. GnRH agonist or synthetic progestins effectively down‐regulate ovarian estradiol biosynthesis and have very little impact on extraovarian estrogens.^7^ In this context, third generation aromatase inhibitors such as anastrozole have been investigated as therapeutic option for women with endometriosis. In cultured endometriotic cells, anastrozole decreases estradiol secretion and endometriotic cell growth.^8^ This molecule has been also shown to reduce endometriosis‐associated pain in small‐scale clinical trials.^7^, ^9^ Although this class of drugs reduces the systemic synthesis of oestrogens, their relevance in the inhibition of the local aromatization within endometriosis implants, as targeted therapy, is still unclear.^4^

In the attempt to investigate anastrozole local effect in human endometriosis, we first investigated a new in‐vivo method allowing the development of human lesions, using chick embryo chorioallantoic membrane (CAM) and then, tested whether blocking local aromatization affects human endometriosis development.

## 2 MATERIALS AND METHODS

### 2.1 Patients and endometriotic tissues

The study was approved by the local Ethics Committee of the University Hospital of Geneva and informed written consent was obtained from all patients. Biopsies of endometriotic ovarian cysts have been collected from women affected by stage III‐IV endometriosis who were undergoing laparoscopy for treatment of endometriosis. At the time of tissue collection, all patients were of reproductive age and none of the women had used hormonal treatment or an intrauterine device within the last 3 months.

Immediately after collection, endometriotic tissues were carefully cut in small fragments in cold Hank's balanced salt solution before being grafted on CAM.

### 2.2 CAM Assay

Fertilized eggs (animal facility of the University of Geneva, Geneva, Switzerland) were incubated with the narrow apex facing downwards and rotated 180° automatically at 37°C and 80% relative humidity. At embryonic development day (EDD), four eggshells were drilled at the narrow apex and adhesive tape was placed on the hole. The eggs were incubated again without rotation, and with the narrow apex upward, for 4 days as previously shown.^10^, ^11^ At EDD8, CAM was gently scratched with a sterile needle close to a blood vessel bifurcation and endometriotic fragments were then graftedEndometriotic ovarian cysts obtained after surgery were cut in several fragments using a biopsy pouch of 3 mm (Fiztmedical Supplies, USA). The window on the eggshell was covered with parafilm and eggs were placed back in the incubator.

At EDD10, lesions were well adherent to CAM and highly vascularized. A silicone O‐ring (Apple Rubber products inc, Lancaster, USA) was placed around the fragment that was topically treated with testosterone (10^‐6^ M, as control), or with anastrozole (1 µg in 30 μL PBS containing 10^‐6^ M testosterone). The eggshell windows were covered with parafilm and eggs replaced in the incubator for 72 hours.

Tissue growth was monitored using a Lumenera INFINITY2‐1 CDD camera with Infinity Capture Software at EDD 10 and 13. Quantitative assessment of tissue growth was measured by ImageJ software.

At EDD13, grafted tissues were excised, washed in PBS, fixed in formalin, dehydrated and fixed in formalin embedded in paraffin (FFPE). Four‐micrometer‐thick sections of (FFPE) CAM tissue were cut and then stained using haematoxylin and eosin (H&E, Sigma‐Aldrich) for histological examination.

### 2.3 Immunohistochemistry

Four‐micrometer tissue sections were deparaffinized and rehydrated through graded ethanol. Antigen retrieval was performed by microwave pre‐treatment in 10 mmol/L citrate buffer (pH 6.0) for 5 minutes four times, followed by cooling in a cold water bath. Non‐specific binding was blocked with 3% (v/v) bovine serum albumin in PBS for 30 minutes at room temperature. Slides were then incubated with anti‐ER–alpha monoclonal primary antibody (1mcg/ml, Roche, Ventana, USA) or monoclonal anti‐aromatase (1:5000, kindly provided by Dr Nobuhiro Harada, Department of Biochemistry, School of Medicine Fujita Health University Toyoake, Japan^12^, ^13^ at 4°C overnight. Sections were then washed with PBS and incubated with appropriate secondary antibodies for 60 minutes at room temperature. After washing, sections were stained with diaminobenzidine chromogen system (Dako, Baar, Switzerlandand) counterstained with hematoxylin (Sigma‐Aldrich). Images of stained sections were captured using EVOS or Olympus BX43 microscope.

### 2.4 Sex determination of chick embryos

Sex of embryos was determined by PCR amplification of a W‐specific *Xho*I repeat from a genomic DNA template following protocol described by Clinton et al.^14^ Amplification of 18S ribosomal DNA was used as PCR control. Chromosomal sex was determined to be ZW (female) if the *Xho*I repeat product was present and ZZ (male) if only the 18S ribosomal repeat product was present. Following dissection of chick embryos, approximately 50 mg of soft tissue was removed and placed in 500 μL of extraction buffer (10 mM Tris‐HCl, 100 mM EDTA, 20 mM NaCl, 1% SDS, pH 8.0 containing 60 μg/mL Proteinase K) and incubated overnight at 45°C, under agitation. Samples were centrifuged at 11 000 rcf for 15 minutes. Supernatants were transferred into clean microcentrifuge tubes and carefully mixed with equivalent volumes of isopropanol. Tubes were centrifuged again at 11 000 rcf for 10 minutes to pellet gDNA. The supernatant were removed and the pellets resuspended in 250 μL of Tris‐EDTA buffer solution. Samples were then incubated overnight at 55°C to allow gDNA to resuspend into solution. gDNA quality and concentration were then evaluated by Nanodrop. Each sample was diluted at 100 ng/μL in sterile water. Primers for W‐specific *Xho*I repeat product detection are: forward: ´CCCAAATATAACACGCTTCACT3´; reverse: 5´AAATGAATTATTTTCTGGCGAC 3´. Primers for 18S ribosomal DNA are: forward: 5´ AGCTCTTTCTCGATTCCGTG 3´ reverse: 3´ GGTAGACACAAGCTGAGCC 3´.

### 2.5 Statistical analysis

Data were expressed as mean ± SEM for n different samples. Statistical analysis of the data was accomplished by the Student's t‐test comparing tissues treated with testosterone alone to those with testosterone plus anastrozole, and comparing these two groups according to the embryo sex. Statistical significance is considered at *P* < 0.05.

## 3 RESULTS

### 3.1 CAM is a suitable model to investigate human endometriosis

**Figure 1:**
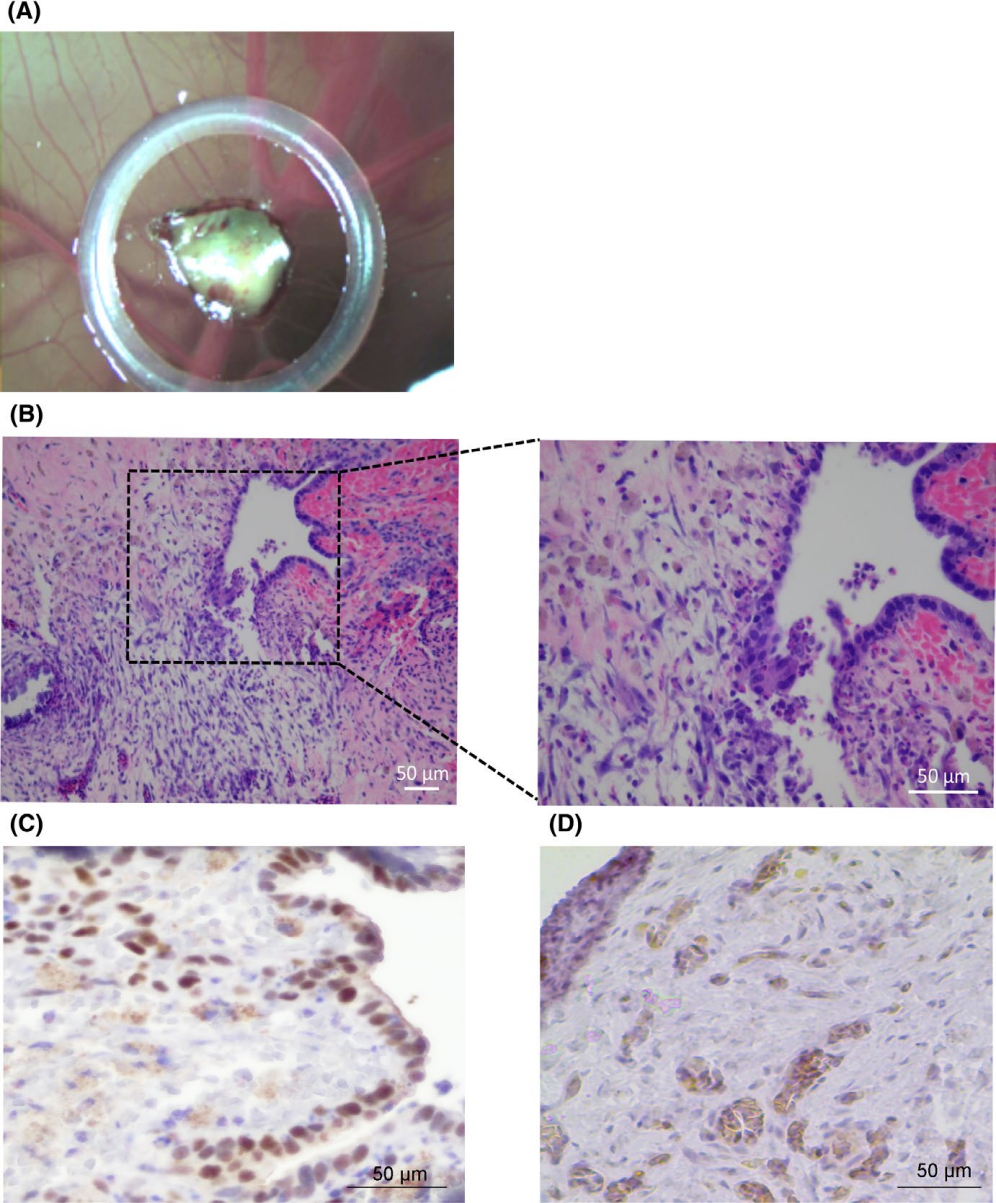
A, Representative image of endometriosis development on xenografted chick embryo chorioallantoic membrane (CAM) at EDD13. B, H&E of endometriosis lesion at EDD13 showing stromal and glandular epithelium (left picture, original magnification X200). C, D, Representative image of immunostaining of ER alpha (C, original magnification X400) and aromatase (D, original magnification X400) in endometriosis cyst on xenografted male CAM

The grafting success of endometriotic tissue was about 80%: after 2 days of incubation, endometriosis lesions were adherent to CAM and highly vascularized (Figure 1A). At EDD13, 5 days after grafting, H&E showed that all lesions cultured onto CAM maintain the characteristics of human endometriosis cyst including stromal and glandular epithelium features (Figure 1B). Immunohistochemistry confirmed positive staining for ER‐alpha (Figure 1C) and aromatase (Figure 1D) in the grafted tissue.

### 3.2 Effects of anastrozole as targeted therapy in endometriosis CAM

**Figure 2:**
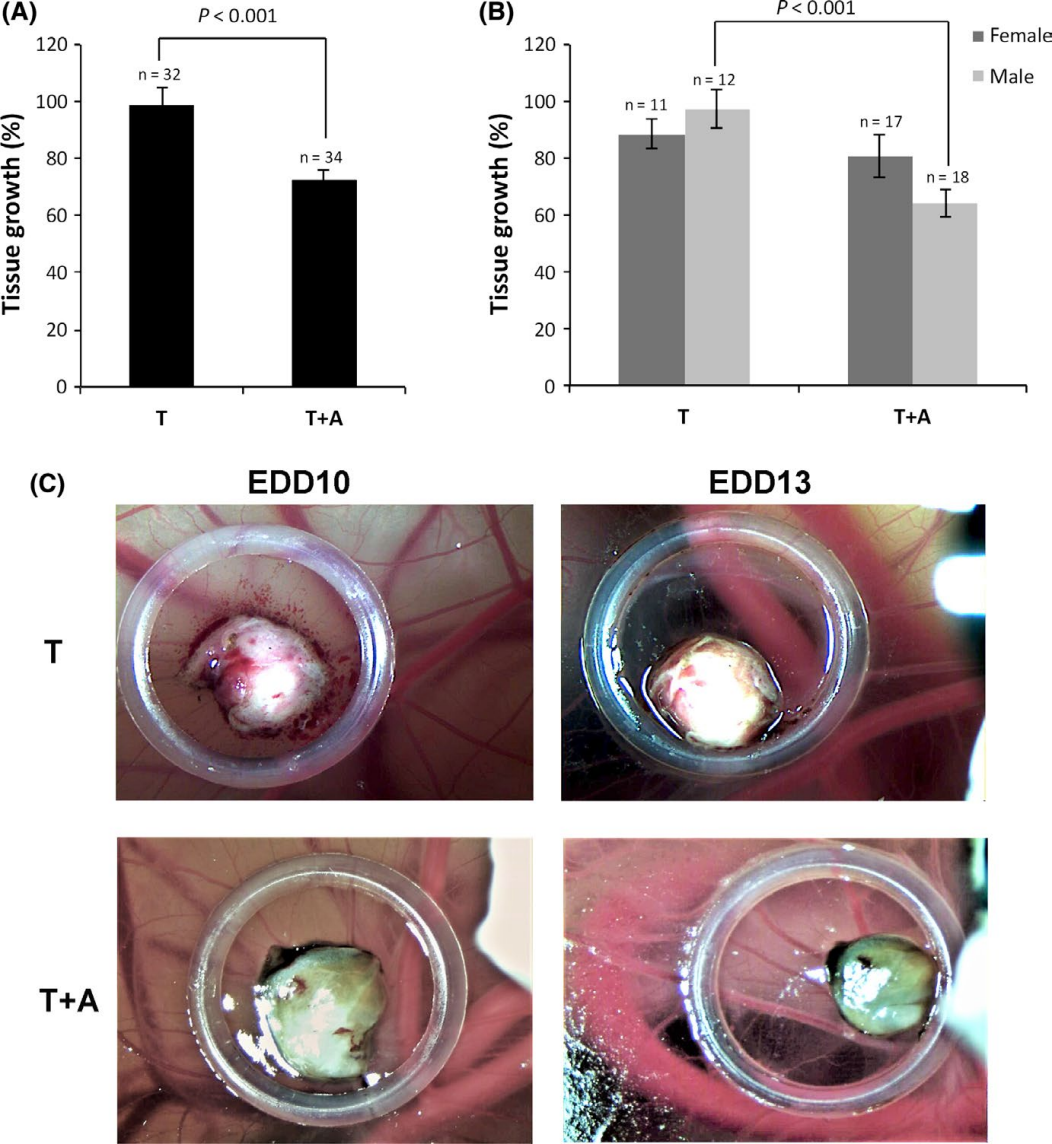
A, Endometriosis growth from EDD10 to EDD13 according to treatment. B, Endometriosis growth changes from EDD10 to EDD13 according to treatment and chick embryo chorioallantoic membrane (CAM) sex. Tissue growth is expressed as percentage (%) of the ratio of lesion sizes measured at EDD10 and at EDD13. Bars represent mean ± SEM. T: testosterone; A: anastrozole. C, Representative images of endometriosis xenografted CAM in the control group and at EDD10 and EDD13

In a first set of experiments, testosterone (T) was topically administered on grafted tissues to provide a substrate for aromatase at EDD10 in combination or not with anastrozole. In the group treated with anastrozole (group A + T, n = 34), the size of implanted endometriotic tissues at EDD13 is significantly decreased by 27% with respect to lesion size before treatment (Figure 2A). In the control group, treated with only testosterone, (group T, n = 32), tissue size at EDD13 was not significantly reduced compared to tissue size before treatment. Therefore, anastrozole significantly decreased endometriotic lesion growth on CAM compared to control group (group T, Figure 2A).

The potential confounding role of endocrine environment (synthesis of oestrogens by female embryos) in endometriosis development and anastrozole effect was evaluated in a second set of experiments.

Based on the knowledge that only female chick embryo starts to synthesize oestrogens at the time of gonadal differentiation (5.5‐6.5 days)^15^ and aromatase is not expressed in male gonads,^16^ treatment groups were analysed according to embryo sex. Anastrozole treatment decreased by 36% the size of grafted tissue in eggs with male embryos compared to controls whereas anastrozole did not show any effect on implanted tissue size in CAM carrying female embryos (Figure 2B,C).

## 4 DISCUSSION

Xenograft CAM is a new efficient model to investigate human endometriosis. This method provides the advantage of working with real endometriotic lesions (endometriotic cyst) with a high rate of tissue xenografting. It allows easy accessibility to ectopic lesions, whose formation can be closely followed during the time course of the experiment. It appears thus to be an appropriate model for the screening of molecules targeting endometriosis for therapeutic purposes.

The CAM assay has already been used as a model for grafting human endometrial tissue^17^, ^18^ with the aim of developing endometriosis. After grafting healthy endometrium, endometriosis‐like lesions were found in 60% of cases.^17^ Here, we used standardized 3 mm biopsies of human endometriosis cyst, as grafted tissue into the CAM. After 5 days following the graft, tissue from all patients maintained endometriosis characteristics at the histological examination. This finding represents a valuable advantage and realistic approach to specifically investigate endometrioma biology from other type of endometriosis and eutopic endometrium.

The present model showed additional interesting findings of CAM biology and specifically in the analysis of the effect of intra‐lesion treatment with anastrozole, as a model of targeted therapy.

Although aromatase inhibitors, combined with progestins or GnRH analogs seem to be effective in suppressing pelvic pain associated with endometriosis, their prolonged use in young premenopausal women is limited by the occurrence of major side effects caused by marked systemic hypooestrogenism.^19^, ^20^ A way to avoid these side effects would be to specifically inhibit aromatase in the endometriosis lesions delivering those molecules into the endometriosis lesions.^21^ However, the relevance in the inhibition of the local aromatization is actually unclear. Here, we observed a significant decrease in endometriosis lesion size following anastrozole topical treatment. However, when we analysed the results according to the embryo sex, topical anastrozole reduced lesion size solely in tissue implanted into CAM carrying a male embryo. Only CAMs carrying female embryos produce oestrogens from EDD5.^16^ Likely, oestrogens produced by the female embryo can reach the grafted endometriotic tissue by systemic circulation and blunting local inhibition of aromatase by anastrozole. In addition, excessive oestrogen signalling of oestrogen receptors of human endometriosis implants 22 may in turn increased local responsiveness to female embryo oestrogens.

Together, these results corroborate the presence of aromatase activity in endometriotic tissue and based on this CAM model, local inhibition of aromatase is not effective in reducing implant size in the presence of a normal estrogenic environment.

CAM is extensively used as a drug‐screening model in cancer biology and angiogenesis.^23^, ^24^ Our findings showed that CAM embryo gender and the resulting endocrine environment during embryo development may represent a cofounding factor, previously underestimated.

In conclusion, CAM is a suitable model for the screening of targeted treatments in endometriosis with less ethical concerns than other animal models.

## CONFLICT OF INTEREST

I certify that neither my co‐authors nor I have a conflict of interest as described above that is relevant to the subject matter or materials included in this work.

## References

[1] Horne AW , Saunders P , Abokhrais IM , Hogg L . Top ten endometriosis research priorities in the UK and Ireland. Lancet. 2017;389:2191‐2192. 10.1016/S0140-6736(17)31344-2 28528751

[2] Burney RO , Giudice LC . Pathogenesis and pathophysiology of endometriosis. Fertil Steril. 2012;98:511‐519. 22819144 10.1016/j.fertnstert.2012.06.029 PMC3836682

[3] Vercellini P , Vigano P , Somigliana E , Fedele L . Endometriosis: pathogenesis and treatment. Nat Rev Endocrinol. 2014;10:261‐275. 24366116 10.1038/nrendo.2013.255

[4] Delvoux B , D'Hooghe T , Kyama C , et al. Inhibition of type 1 17beta‐hydroxysteroid dehydrogenase impairs the synthesis of 17beta‐estradiol in endometriosis lesions. J Clin Endocrinol Metab. 2014;99:276‐284. 24187399 10.1210/jc.2013-2503

[5] Attar E , Bulun SE . Aromatase and other steroidogenic genes in endometriosis: translational aspects. Hum Reprod Update. 2006;12:49‐56. 16123052 10.1093/humupd/dmi034

[6] Bulun SE , Nezhat C . Aromatase, microRNA, and inflammation: a complex relationship. Fertil Steril. 2016;106:552‐553. 27473353 10.1016/j.fertnstert.2016.06.045 PMC5010923

[7] Pluchino N , Freschi L , Wenger JM , Streuli I . Innovations in classical hormonal targets for endometriosis. Expert Rev Clin Pharmacol. 2016;9:317‐327. 26645363 10.1586/17512433.2016.1129895

[8] Badawy SZ , Brown S , Kaufman L , Wojtowycz MA . Aromatase inhibitor (anastrozole) affects growth of endometrioma cells in culture. Eur J Obstet Gynecol Reprod Biol. 2015;188:45‐50. 25839436 10.1016/j.ejogrb.2015.01.009

[9] Ferrero S , Gillott DJ , Venturini PL , Remorgida V . Use of aromatase inhibitors to treat endometriosis‐related pain symptoms: a systematic review. Reprod Biol Endocrinol. 2011;9:89. 21693038 10.1186/1477-7827-9-89 PMC3141646

[10] Cohen M , Pierredon S , Wuillemin C , Delie F , Petignat P . Acellular fraction of ovarian cancer ascites induce apoptosis by activating JNK and inducing BRCA1, Fas and FasL expression in ovarian cancer cells. Oncoscience. 2014;1:262‐271. 25594018 10.18632/oncoscience.31 PMC4278302

[11] Meynier S , Kramer M , Ribaux P , et al. Role of PAR‐4 in ovarian cancer. Oncotarget. 2015;6:22641‐22652. 26246468 10.18632/oncotarget.4010 PMC4673188

[12] Sasano H , Frost AR , Saitoh R , et al. Aromatase and 17 beta‐hydroxysteroid dehydrogenase type 1 in human breast carcinoma. J Clin Endocrinol Metab. 1996;81:4042‐4046. 8923858 10.1210/jcem.81.11.8923858

[13] Utsunomiya H , Suzuki T , Kaneko C , et al. The analyses of 17beta‐hydroxysteroid dehydrogenase isozymes in human endometrial hyperplasia and carcinoma. J Clin Endocrinol Metab. 2001;86:3436‐3443. 11443221 10.1210/jcem.86.7.7661

[14] Zhao D , McBride D , Nandi S , et al. Somatic sex identity is cell autonomous in the chicken. Nature. 2010;464:237‐242. 20220842 10.1038/nature08852 PMC3925877

[15] Vaillant S , Dorizzi M , Pieau C , Richard‐Mercier N . Sex reversal and aromatase in chicken. J Exp Zool. 2001;290:727‐740. 11748621 10.1002/jez.1123

[16] Kamata R , Takahashi S , Morita M . Gene expression of sex‐determining factors and steroidogenic enzymes in the chicken embryo: influence of xenoestrogens. Gen Comp Endocrinol. 2004;138:148‐156. 15302264 10.1016/j.ygcen.2004.05.011

[17] Maas JW , Groothuis PG , Dunselman GA , de Goeij AF , Struijker‐Boudier HA , Evers JL . Development of endometriosis‐like lesions after transplantation of human endometrial fragments onto the chick embryo chorioallantoic membrane. Hum Reprod. 2001;16:627‐631. 11278208 10.1093/humrep/16.4.627

[18] Malik E , Meyhofer‐Malik A , Berg C , et al. Fluorescence diagnosis of endometriosis on the chorioallantoic membrane using 5‐aminolaevulinic acid. Hum Reprod. 2000;15:584‐588. 10686200 10.1093/humrep/15.3.584

[19] Committee opinion no. 663 summary: aromatase inhibitors in gynecologic practice. Obstet Gynecol. 2016;127:1187‐1188. 27214185 10.1097/AOG.0000000000001478

[20] Dunselman GA , Vermeulen N , Becker C , et al. ESHRE guideline: management of women with endometriosis. Hum Reprod. 2014;29:400‐412. 24435778 10.1093/humrep/det457

[21] Friend DR . Drug delivery for the treatment of endometriosis and uterine fibroids. Drug Deliv Transl Res. 2017;7:829‐839. 28828592 10.1007/s13346-017-0423-2

[22] Zhao Y , Chen Y , Kuang Y , et al. Multiple beneficial roles of repressor of estrogen receptor activity (REA) in suppressing the progression of endometriosis. Endocrinology. 2016;157:900‐912. 26653759 10.1210/en.2015-1324 PMC4733120

[23] Ribatti D . The chick embryo chorioallantoic membrane (CAM). A multifaceted experimental model. Mech Dev. 2016;141:70‐77. 27178379 10.1016/j.mod.2016.05.003

[24] Zuo Z , Syrovets T , Wu Y , et al. The CAM cancer xenograft as a model for initial evaluation of MR labelled compounds. Sci Rep. 2017;7:46690. 28466861 10.1038/srep46690 PMC5413881

