# Supplementary figures and images for: Effect of local aromatase inhibition in endometriosis using a new chick embryo chorioallantoic membrane model
Source: J Cell Mol Med. 2019 Jun 14;23(8):5808–12. doi: 10.1111/jcmm.14372 (PMC6653393; doi:10.1111/jcmm.14372)

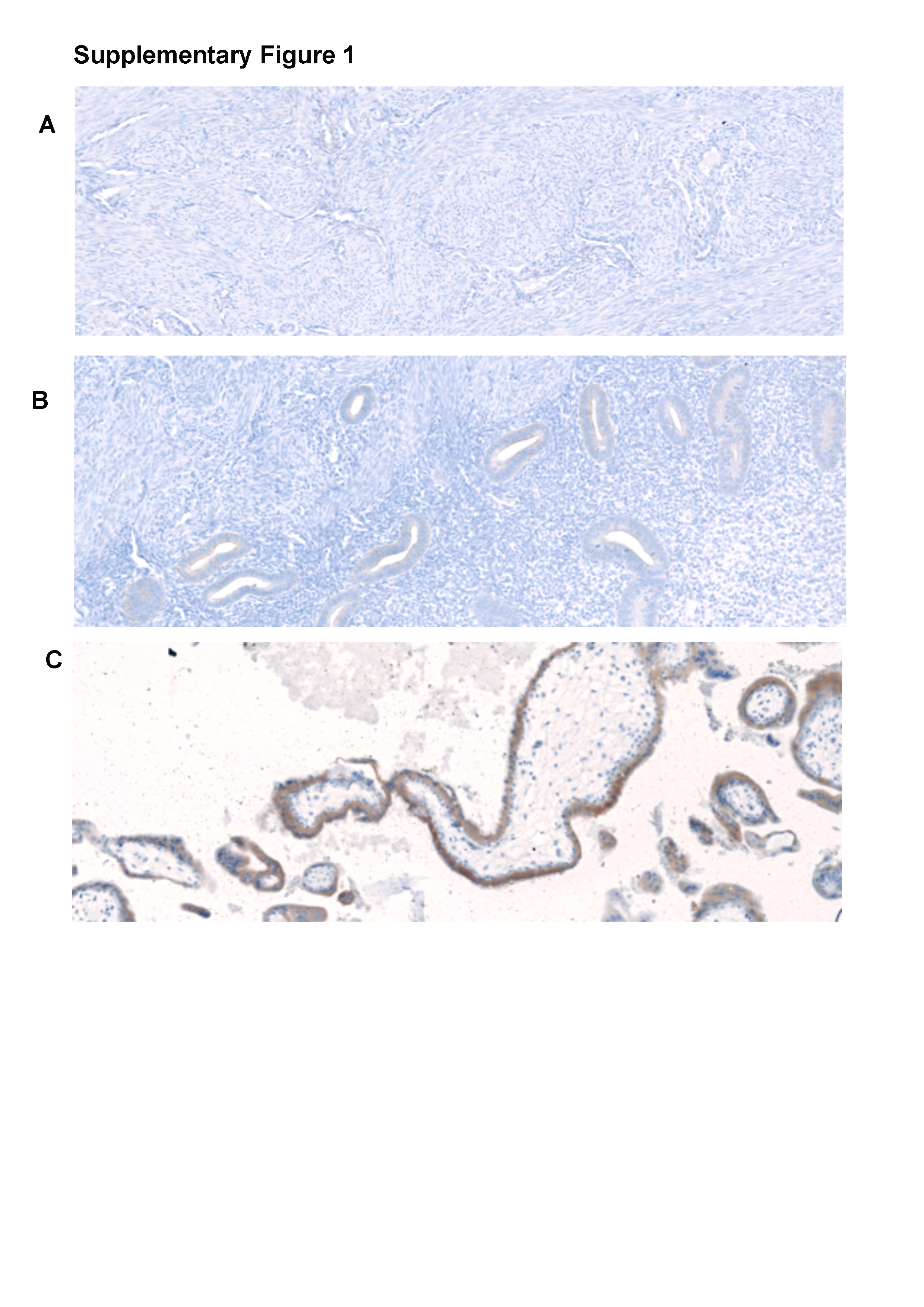

Supplement: Supplementary file 1 [file JCMM-23-5808-s001.tif]
